# Ubiquitin-centered post-translational modification crosstalk orchestrates tumor immunity and immunotherapy response

**DOI:** 10.1186/s40164-026-00754-8

**Published:** 2026-02-08

**Authors:** Kailin Qiao, Leilei Wu, Letong Yang, Ming Liu, Chenxue Jiang, Yun Chen, Zhenshan Zhang, Jinming Yu, Dongping Wei, Yaping Xu

**Affiliations:** 1 Department of Radiation Oncology, Shanghai Pulmonary Hospital, School of Medicine, Tongji University, No.507 Zhengmin Road, Yangpu District, Shanghai, 200433 China; 2 Department of Thoracic Surgery, Shanghai Pulmonary Hospital, School of Medicine, Tongji University, Shanghai, 200433 China; 3 Department of Radiation Oncology, Shandong Cancer Hospital and Institute, Shandong Academy of Medical Sciences, Shandong First Medical University, Jinan, 250117 Shandong China; 4 Medical Research Center, The First Affiliated Hospital of Wenzhou Medical University, Wenzhou, 325015 Zhejiang China

**Keywords:** Ubiquitination regulation, Tumor immunotherapy, Tumor immune microenvironment, Immune checkpoint, Targeted therapy

## Abstract

Remarkable progress has been made in cancer immunotherapy in recent years; however, it still faces challenges such as limited response rates, resistance, and immune-related adverse events. Ubiquitination, a key post-translational modification (PTM) of proteins, is indispensable for regulating various tumor immunity-related processes. Through the dynamic balance between ubiquitin ligases and deubiquitinases, this PTM fine-tunes the strength and duration of immune responses, influencing tumor recognition and immune evasion. Accumulating evidence reveals that ubiquitination does not act alone but cooperates and competes with other PTMs—such as phosphorylation, acetylation, SUMOylation, neddylation, and glycosylation—to form a multilayered regulatory network that determines the immune landscape and therapeutic responsiveness. This review systematically summarizes the molecular mechanisms by which ubiquitination-related enzymes modulate the tumor immune microenvironment and immune evasion. Moreover, we highlight emerging insights into the crosstalk between ubiquitination and other PTMs, which collectively govern the stability and signaling of immune regulators. Finally, we discuss the translational potential of targeting the ubiquitin system, emphasizing opportunities and challenges in developing selective ubiquitin modulators and designing rational combination immunotherapies. Decoding this integrated PTM network will not only deepen mechanistic understanding of tumor immunity but also open new avenues for precision immunotherapy.

## Introduction

Cancer remains one of the most significant public health challenges worldwide, resulting in nearly one-sixth of all deaths worldwide [1]. Under physiological homeostasis, cell growth is regulated by various signals. However, when certain cellular mechanisms such as the cell cycle, signal transduction, or apoptosis become dysregulated, cancer can develop [2]. One of the hallmarks of cancer is its ability to evade immune destruction [3]. Immune checkpoints are composed of a series of molecules presented on the membranes of immune cells. These molecules act as regulators of immune activation and are vital for the regulation of self-tolerance and the prevention of immune system hyperactivation [4, 5]. However, tumor cells often exploit these checkpoints to avoid recognition by the immune system, making them key focuses of cancer immunotherapy studies [6]. In recent years, with the increasing understanding of the relationship between tumor cells and the tumor immune microenvironment (TIME), cancer immunotherapy has attracted significant interest from researchers and has profoundly transformed the field of cancer therapy [7]. The emergence of immune checkpoint inhibitors (ICIs), particularly programmed cell death protein 1/programmed cell death ligand 1 (PD-1/PD-L1) and cytotoxic T-lymphocyte-associated protein 4 (CTLA-4) blockers, has made immunotherapy a standard treatment modality for several advanced cancers, improving the prognosis of cancer patients. However, current immunotherapies face bottlenecks and challenges, as the overall response rate to ICIs is not ideal. The persistence of resistance, including both primary and acquired resistance, remains a significant barrier in the realm of cancer immunotherapy [8]. Therefore, acquiring a more profound comprehension of the regulatory mechanisms within the tumor microenvironment (TME) and antitumor immunity is crucial.

**Fig. 1:**
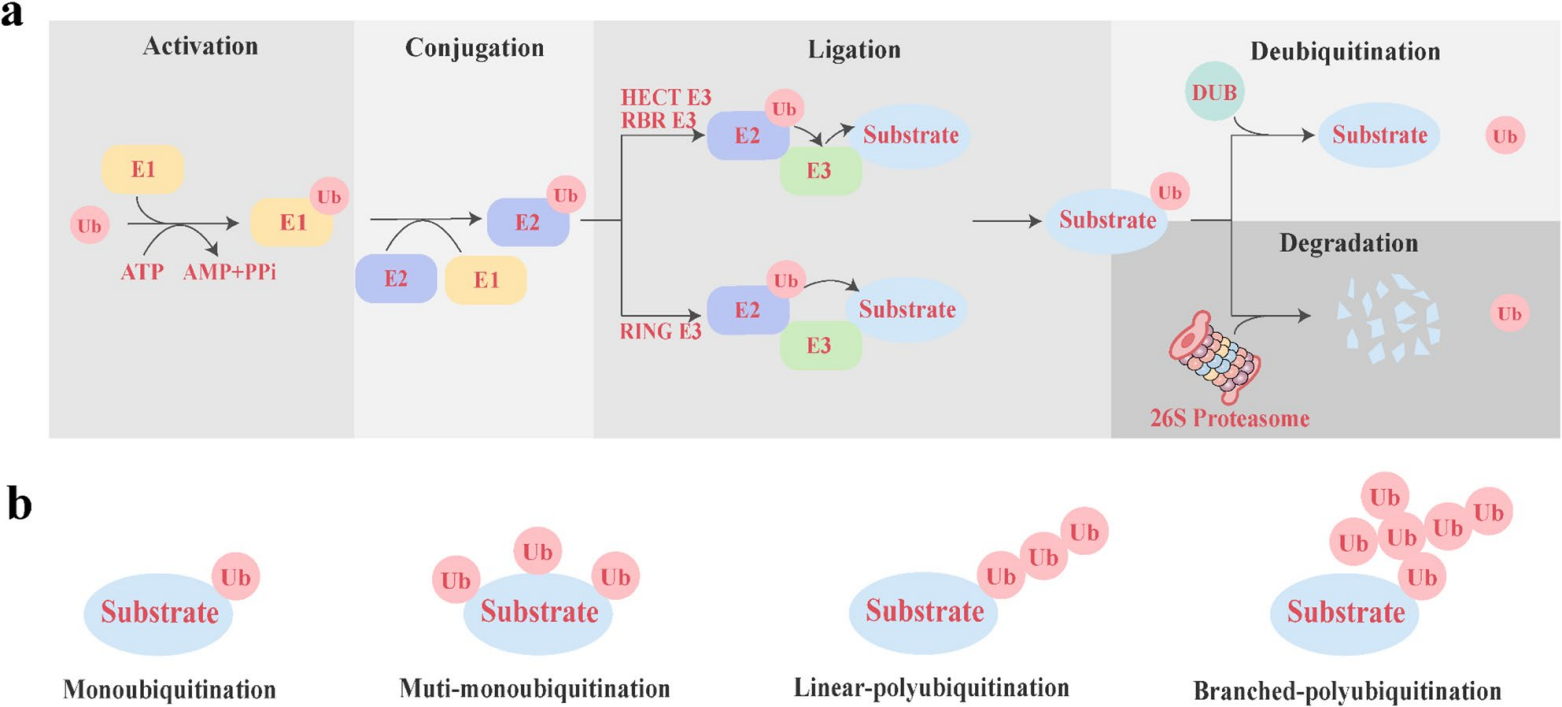
Overview of the ubiquitination process. **a** Mechanisms of the ubiquitin–proteasome system: activation, conjugation, ligation, deubiquitination and degradation. **b** Different types of ubiquitination: monoubiquitination, multi-monoubiquitination, linear-polyubiquitination and branched- polyubiquitination. *Ub* ubiquitin, *ATP* adenosine triphosphate, *AMP* adenosine monophosphate, *E1* ubiquitin-activating enzymes, *E2* ubiquitin-conjugating enzymes, *E3* ubiquitin ligases, *RING* Really Interesting New Gene, *HECT* Homologous to E6-AP Carboxyl Terminus, *RBR* RING-Between-RING, *DUB* deubiquitinating enzyme

Epigenetic reprogramming and post-translational modifications (PTMs) of proteins, such as ubiquitination, phosphorylation, and acetylation, are closely associated with tumorigenesis, cancer progression, and immune regulation [9]. Targeting the enzymes responsible for these modifications has been demonstrated to effectively induce cell death and suppress tumor growth [10]. It is important to note that these PTMs do not work in isolation, but crosstalk with each other to form a complex network, which plays an important role in tumor immune response. Ubiquitination is an ATP-dependent, highly specific process in which substrate proteins are tagged with ubiquitin through a cascade of reactions [11]. Ubiquitin is a small protein consisting of 76 amino acids [12]. Ubiquitination, along with protein degradation by the proteasome, involves the ubiquitin-proteasome system (UPS), which is responsible for 80–90% of cellular protein hydrolysis and 10–20% of autophagy [13]. Usually, ubiquitination degrades substrates via the K48-linked chain, whereas the K63-linked chain is associated primarily with non-proteasomal degradation processes [14]. Ubiquitination proceeds through sequential steps mediated by ubiquitin-activating enzymes (E1), ubiquitin-conjugating enzymes (E2), and ubiquitin ligases (E3) [15]. Initially, the E1 enzyme activates ubiquitin in an ATP-dependent process. The activated ubiquitin is passed to the E2 enzyme by forming a thioester bond. Then, the E3 ligase promotes the covalent linkage of ubiquitin to lysine residues on the target protein [16, 17] (Fig. 1a). E3 provides substrate specificity and is categorized into three major types on the basis of its catalytic domain characteristics and the mechanism of ubiquitin transfer to the substrate: RING (Really Interesting New Gene), HECT (Homologous to E6-AP Carboxyl Terminus), and RBR (RING-Between-RING) ligases [12]. Ubiquitin molecules can either be monoubiquitinated at any one of seven internal lysine residues (Lys6, Lys11, Lys27, Lys29, Lys33, Lys48, or Lys63) or assemble into polyubiquitin chains on the target substrate [18] (Fig. 1b). Ubiquitination is reversible, and the reverse process is called deubiquitination. Deubiquitination carried out by deubiquitinating enzymes (DUBs), which remove ubiquitin chains and hydrolyze them into individual ubiquitin molecules [19]. Ubiquitination and deubiquitination play critical regulatory roles in tumorigenesis and cancer progression, influencing cell survival, proliferation, and differentiation. They are also widely involved in the stability of immune checkpoints and the regulation of antitumor immune activity, offering significant potential for enhancing the efficacy of immunotherapy [20–22].

In this review, we first introduce the current challenges faced by cancer immunotherapy. Next, we summarize the roles of ubiquitination-related mechanisms in regulating the TIME and tumor immune evasion, highlighting how ubiquitination and its crosstalk with other PTMs shape tumor immunity. Finally, we discuss the future prospects and challenges of targeting ubiquitination regulatory network in cancer therapy.

## Challenges in cancer immunotherapy

**Fig. 2:**
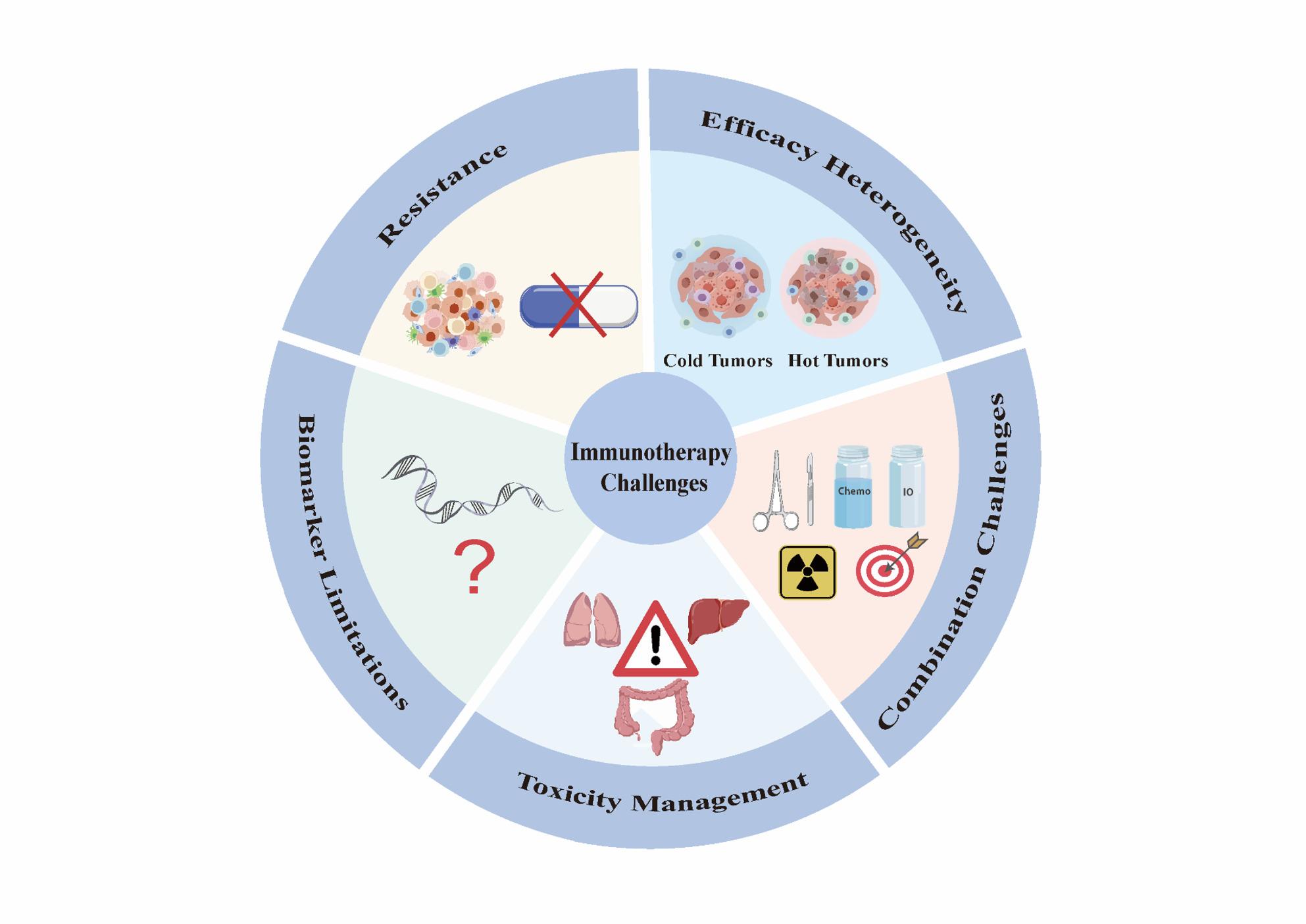
Immunotherapy challenges: efficacy heterogeneity, combination challenges, toxicity management, biomarker limitations and resistance

Cancer immunotherapy represents a therapeutic approach that harnesses the cytotoxic capabilities of the human immune system, particularly tumor-specific cytotoxic T cells, to treat malignancies. In general, cancer immunotherapy can be categorized into two types [23, 24]: active immunotherapy, such as ICIs [25–27], cytokine therapy [28], and cancer vaccines [29–31], and passive immunotherapy, such as chimeric antigen receptor T cells (CAR-T cells) therapy [32] and anti-tumor monoclonal antibodies [33]. Among the diverse cancer immunotherapies, ICIs currently have the most profound influence and have achieved the greatest degree of clinical success. However, current cancer immunotherapy methods face multiple challenges, including but not limited to: heterogeneous therapeutic efficacy (e.g., immunologically “cold” versus “hot” tumor phenotypes), resistance mechanisms (both primary and acquired resistance), management of immune-related adverse events (irAEs), insufficient predictive capacity of biomarkers and poorly defined synergistic mechanisms of combination therapies [34–38] (Fig. 2).

The response rates to immunotherapy differ markedly among cancer types [39, 40], with response rates in many tumor types ranging from 20% to 40% [41]. Only a small number of patients with advanced cancer achieve long-term survival from these therapies. Even in melanoma patients, who exhibit relatively high response rates to ICIs, effective and durable responses are limited to a small subset of patients, with 60–70% showing no objective response, i.e., primary resistance. Additionally, patients who initially respond well may develop resistance over time, known as acquired resistance, with 20–30% of patients eventually experiencing tumor recurrence and progression, necessitating a change in treatment strategy [42, 43]. Therefore, resistance or non-responsiveness to immunotherapy remains a critical issue. These outcomes likely reflect the complex and highly regulated nature of the immune system. Intrinsic factors of tumor cells (including the modulation of tumor cell–intrinsic genes and signaling pathways that obstruct immune cell infiltration or functionality in the TME) as well as components of the TME other than tumor cells (such as regulatory T [Treg] cells, myeloid-derived suppressor cells [MDSCs], M2 macrophages, and other inhibitory immune checkpoints) may contribute to the suppression of anti-tumor immune responses, leading to immunotherapy resistance [44]. Thus, there is a need for a better understanding and modulation of the multiple factors that influence anti-tumor immune responses to increase the proportion of patients who benefit from cancer immunotherapy.

## Ubiquitination process regulates TIME

The TIME refers to the complicated system of immune cells, stromal components, signaling molecules, and the extracellular matrix that surrounds and infiltrates tumor tissues [45]. These components interact with tumor cells to either promote immune-mediated tumor clearance or support immune evasion and tumor progression. The TIME plays a central role in determining tumor behavior, response to immunotherapy, and clinical outcome [46]. Ubiquitination-related processes can regulate various immune cells and signaling molecules within the TIME, and are essential for identifying novel therapeutic targets and enhancing the efficacy of cancer immunotherapy.

### Ubiquitination process regulates immune cells

#### T cells

**Fig. 3:**
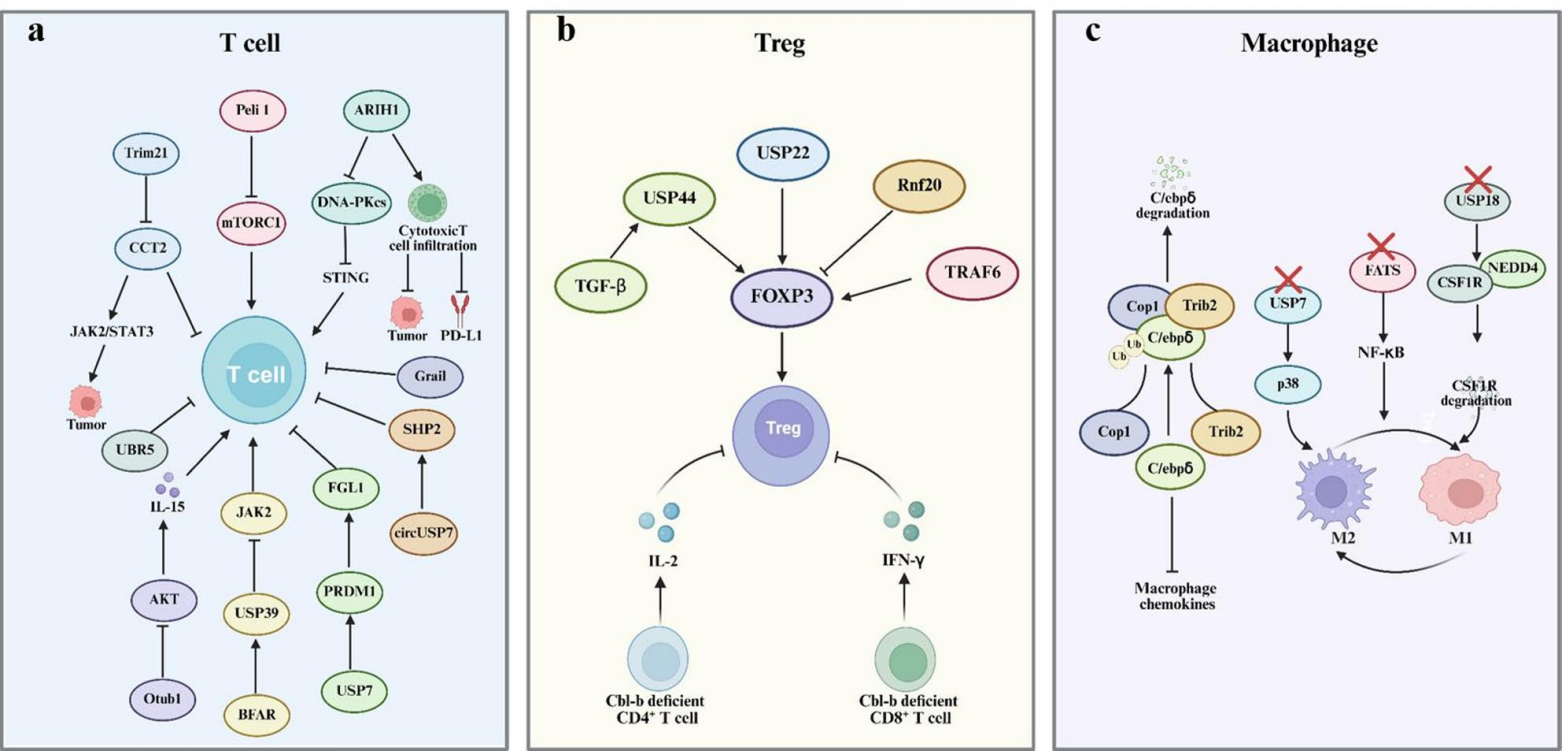
The ubiquitination process regulates immune cells. **a** The ubiquitination process regulates T cells. **b** The ubiquitination process regulates Tregs. **c** The ubiquitination process regulates macrophages. *ARIH1* ariadne RBR E3 ubiquitin‑protein ligase 1, *PD-L1* programmed cell death ligand 1, *UBR5* ubiquitin protein ligase E3 component n‑recognin 5, *USP* ubiquitin-specific protease, *PRDM1* PR domain zinc finger protein 1, *SHP2* SH2-containing protein tyrosine phosphatase 2, *TGF-β* transforming growth factor-β, *RNF* Ring finger protein, *FOXP3* forkhead box P3, *TRAF6* TNF receptor‑associated factor 6, *Treg* regulatory T, *IL* interleukin, *Ub* ubiquitin, *FATS* fragile site-associated tumor suppressor, *NEDD4* neural precursor cell expressed developmentally downregulated 4

The regulation of T cell activity through ubiquitination and deubiquitination mechanisms plays a key role in modulating tumor immunity, highlighting the potential of targeting these pathways for cancer immunotherapy. Various studies have identified key players in this process, suggesting promising therapeutic strategies. Ariadne RBR E3 ubiquitin‑protein ligase 1 (ARIH1) catalyzes the ubiquitination and subsequent degradation of DNA‑PKcs, thereby triggering STING pathway activation and promoting T cell activation. The overexpression of *Arih1* enhances cytotoxic T cell infiltration, suppresses tumor growth, and potentiates the efficacy of PD‑L1 blockade. ACY738 upregulates ARIH1 and activates STING signaling, sensitizing tumors to PD‑L1 inhibition [47]. CCT2 can inhibit CD4^+^ T cell activation and pro‑inflammatory cytokine secretion in breast cancer, and can promote tumor growth and metastasis through the JAK2/STAT3 signaling pathway. The E3 ubiquitin ligase Trim21 can mediate the ubiquitination of CCT2, suppress the malignant progression of breast cancer, and enhance CD4^+^ T cell activation [48]. As an essential E3 ubiquitin ligase, Peli1 orchestrates T cell metabolism and the immune response against tumors. Peli1 deletion markedly promotes tumor rejection, accompanied by increased infiltration of CD4⁺ and CD8⁺ T cells. Peli1 regulates activation of the metabolic kinase mTORC1—stimulated by both TCR signaling and growth factors—by modulating the mTORC1‑inhibitory proteins TSC1 and TSC2. Peli1 catalyzes non‑degradative ubiquitination of TSC1, thereby facilitating TSC1–TSC2 dimerization and stabilizing TSC2 [49]. Ubiquitin protein ligase E3 component n‑recognin 5 (UBR5, also known as EDD) belongs to the HECT‑domain E3 ubiquitin ligase family. It promotes tumor growth mainly via paracrine modulation of the immune microenvironment, chiefly by suppressing CD8⁺ T lymphocyte–driven cytotoxic responses. Targeting UBR5 can induce CD8^+^ T cell-mediated immune response, which is of significant importance for the development of new immunotherapies [50]. The expression of the E3 ubiquitin ligase Grail is elevated in CD8⁺ T cells infiltrating transplanted lymphoma tumors, and Grail deficiency enables durable tumor control. Loss of Grail bolsters the antitumor activity and functional capacity of CD8⁺ T cells. Moreover, Grail‑deficient CD8⁺ T cells exhibit upregulated interleukin (IL)‑21 receptor (IL‑21R) expression and heightened responsiveness to IL‑21 signaling, since Grail normally mediates IL‑21R ubiquitination and degradation [51]. CD8^+^ T cells and natural killer (NK) cells rely on IL-15 to maintain homeostasis in vivo. Otub1, a deubiquitinase, regulates CD8⁺ T cell and NK cell activation by functioning as an IL‑15–dependent checkpoint [52]. The E3 ubiquitin ligase BFAR is markedly upregulated in aged CD8⁺ T cells and has been recognized as an inhibitory regulator of tissue-resident memory T_RM_ cell differentiation. Mechanistically, BFAR promotes the deubiquitination of JAK2 by activating the deubiquitinase ubiquitin-specific protease 39 (USP39), which in turn suppresses STAT1 phosphorylation—a key signaling event required for CD103 expression and T_RM_ generation. This suppression impairs the formation and effector function of CD8⁺ T_RM_ cells in the TME [53]. By deubiquitinating the transcription factor PR domain zinc finger protein 1 (PRDM1), USP7 enhances FGL1 expression, which in turn suppresses CD8⁺ T cell activity and promotes liver cancer progression. The USP7 inhibitor P5091 can downregulate FGL1 expression, enhance CD8^+^ T cell activity, and has potential as a target for liver cancer immunotherapy [54]. In non-small cell lung cancer (NSCLC) patient plasma, circUSP7 is predominantly secreted by NSCLC cells via exosomes and suppresses CD8^+^ T cell secretion of IFN-γ, TNF-α, Granzyme‑B, and perforin. Furthermore, by sponging miR‑934, circUSP7 upregulates Src homology region 2 (SH2)-containing protein tyrosine phosphatase 2 (SHP2) expression, further inhibiting CD8^+^ T cell function. CircUSP7 also induces resistance to anti‑PD1 immunotherapy, representing a promising therapeutic approach for individuals with NSCLC [55] (Fig. 3a).

#### Treg

Treg cells play crucial roles in controlling immune responses and preserving immune homeostasis. However, they also represent a significant barrier to effective antitumor immunity. Treg instability is marked by the loss of the master transcription factor forkhead box P3 (FOXP3) and the acquisition of proinflammatory properties. Sustained FOXP3 expression is essential for the ability of Tregs to preserve immunological self-tolerance through their suppressive activity. Several regulators influence the stability of FOXP3 and, consequently, Treg function. USP22, a member of the SAGA chromatin-modifying complex, stabilizes FOXP3 expression, whereas Rnf20, an E3 ubiquitin ligase, acts as an inhibitory regulator of FOXP3. These findings suggest that USP22 and Rnf20 could be potential targets for Treg-based immunotherapy [56]. Additionally, USP44, a deubiquitinase, stabilizes FOXP3 by removing K48-linked ubiquitin modifications. Transforming growth factor-β (TGF-β) induces USP44 expression during Treg differentiation, further highlighting its role in regulating Treg stability [57]. K63‑linked ubiquitination by TNF receptor‑associated factor 6 (TRAF6) directs FOXP3 to its correct cellular compartment and enhances its transcriptional regulatory function in Tregs. Thus, TRAF6 serves as a pivotal post‑translational modulator of Treg stability and represents a potential target for novel tolerance‑disrupting therapies [58]. Moreover, the E3 ubiquitin ligase Cbl-b has been shown to establish resistance to Treg-mediated suppression. Cbl-b deficiency in CD4^+^ T cells results in overproduction of IL-2, and in CD8^+^ T cells, overproduction of IFNγ, both of which confer resistance against Treg suppression [59, 60]. These findings suggest that targeting Cbl-b could enhance immune responses by overcoming Treg-mediated inhibition. In summary, targeting the regulators of FOXP3 stability offers promising strategies to modulate Treg function and improve antitumor immunity (Fig. 3b).

#### Macrophage

Macrophages are essential immune cells that play pivotal roles in tumor immunity [61]. Depending on the microenvironment, macrophages can be classified into two main subtypes: classical M1 polarization and alternative M2 polarization [62]. Multiple studies have confirmed that the processes of ubiquitination and deubiquitination play crucial roles in regulating macrophages. In triple-negative breast cancer (TNBC), via the adaptor protein Trib2, the E3 ubiquitin ligase Cop1 controls C/ebpδ protein levels, and C/ebpδ in turn transcriptionally inhibits cancer cell release of macrophage chemoattractants. The deletion of Cop1 reduces the secretion of macrophage-associated chemokines, decreases tumor macrophage infiltration, enhanced anti-tumor immunity, and improves the response to immune checkpoint blockade [63]. The E3 ubiquitin-conjugating enzyme fragile site-associated tumor suppressor (FATS) plays a crucial role in tumor development by regulating tumor immunity. FATS deficiency drives M1 polarization by disrupting the NF‑κB/IκBα negative feedback loop, thereby stimulating and sustaining NF‑κB activation. This reprograms tumor‑associated macrophages from an M2‑like, tumor‑promoting phenotype to an M1‑like, antitumor phenotype, ultimately inhibiting tumor growth. Thus, FATS functions as an immune regulator and represents a promising target for cancer immunotherapy [64]. USP7 is highly expressed in M2 macrophages, and targeted inhibition of USP7 can induce the polarization of M2 macrophages toward the M1 phenotype, suppress their function, and activate the p38 MAPK pathway. In vivo, it can also modulate the TIME, affect tumor growth, and synergize with immunotherapy, making it of significant importance in lung cancer treatment [65]. Similarly, USP18 plays a crucial regulatory role in macrophages. It inhibits the binding of the ubiquitin E3 ligase neural precursor cell expressed developmentally downregulated 4 (NEDD4) to CSF1R, thereby blocking the ubiquitination and subsequent degradation of CSF1R, which helps maintain CSF1R levels and influences macrophage differentiation and polarization. Additionally, its deficiency enhances IFN-I signaling, promoting the increased expression of the IFN-I-induced ubiquitin E2 enzyme UBCH5. This, in turn, cooperates with NEDD4 to increase the degradation of CSF1R, ultimately driving the polarization of tumor-associated macrophages (TAMs) toward an antitumor/pro-inflammatory phenotype [66]. This alteration of the cellular composition and function within the TME significantly impacts tumor progression and provides a potential new therapeutic target and theoretical basis for cancer treatment (Fig. 3c).

### Ubiquitination process regulates signaling molecules

#### TGF-β

**Fig. 4:**
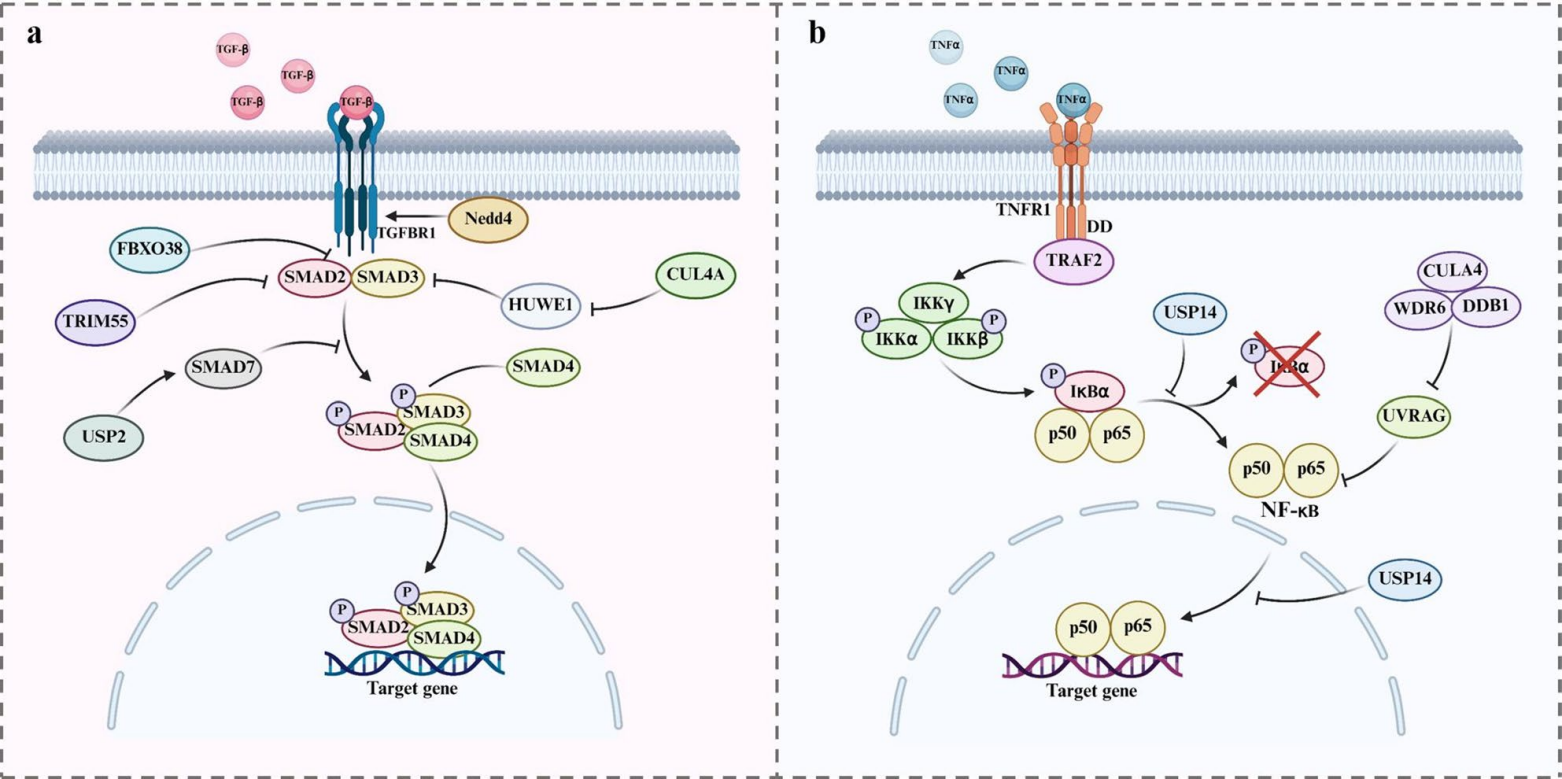
The ubiquitination process regulates TGF-β (**a**) and TNFα (**b**). *TGF-β* transforming growth factor-β, *TNFα* tumor necrosis factor α, *NEDD4* neural precursor cell expressed developmentally downregulated 4, *TGFBR1* TGF-β type I receptor, *USP* ubiquitin-specific protease, *TRAF* TNF receptor‑associated factor, *DD* death domain, *IKK* inhibitor of kappa B kinase, *IκBα* inhibitor of kappa B alpha

TGF-β plays a crucial regulatory role in tumor survival, proliferation, metastasis, and functions within the TME [67]. TGF-β-triggered signaling can be regulated by ubiquitination and deubiquitination. Several E3 ligases and deubiquitinases tightly regulate the TGF-β pathway. SMAD7 functions as a primary suppressor of TGF‑β signaling. USP2 can deubiquitinate SMAD7 by directly cleaving the Lys27- and Lys48-linked polyubiquitin chains on SMAD7, thereby suppressing the TGF-β signaling pathway [68]. The E3 ligase NEDD4 is associated with the progression of hepatocellular carcinoma (HCC). Knockout of *Nedd4* can inhibit HCC metastasis and in vivo growth. Mechanistically, NEDD4 promotes tumor progression mediated by TGF-β signaling by directly binding to the TGF-β type I receptor (TGFBR1) and catalyzing K27-linked ubiquitination at lysine 391 [69]. As the core scaffold protein of the Cullin‑RING E3 ligases 4 (CRL4) complex, CUL4A can regulate the level of TGF-β and has dual roles in colorectal cancer. A previous study demonstrated that CUL4A negatively regulates the stability of HUWE1, thereby affecting the ubiquitination status and intracellular localization of SMAD3, a key transcription factor in the TGF-β signaling pathway [70]. Loss of CUL4A leads to the accumulation of HUWE1, which enhances the ubiquitination of SMAD3 and promotes its retention in the cytoplasm, thereby weakening TGF-β signaling and ultimately contributing to tumor initiation and progression. FBXO38, an E3 ubiquitin ligase, has been identified as a key regulator of NK cell–mediated antitumor immunity by modulating TGF-β signaling within the TME. In tumor-infiltrating NK (TINK) cells, FBXO38 counteracts the TGF-β-induced suppression of IL-15 receptor (IL-15R) expression by limiting Smad2/3 signaling and preserving the expression of Eomes, a transcription factor essential for IL-15R transcription [71]. These findings highlight the role of FBXO38-mediated ubiquitination in mitigating TGF-β–induced immunosuppression and sustaining NK-cell responsiveness to IL-15 within the TME. TRIM55 promotes the degradation of the double-stranded RNA-binding protein NF90 via the ubiquitin–proteasome pathway in HCC, thereby disrupting NF90-mediated stabilization of the HIF1α and TGF-β2 mRNAs. This leads to reduced HIF1α protein expression and VEGF secretion, and inhibits TGF-β2–induced Smad2 phosphorylation. As a result, TRIM55 suppresses HIF1α/VEGF-mediated angiogenesis and TGF-β/Smad signaling–driven immunosuppression [72] (Fig. 4a).

#### Tumor necrosis factor α (TNFα)

TNFα is an inflammatory cytokine that induces cell death via TNF receptors (TNFR) through extrinsic caspase‑ or necroptosis‑dependent pathways, as well as through intrinsic reactive oxygen species– and DNA damage–mediated mechanisms [73]. Its cytotoxic effects are frequently counteracted by activation of the NF‑κB signaling cascade, a central regulator of cell survival and therapeutic resistance in multiple cancers [74]. TRAF2 is a bifunctional protein that serves both as an adaptor and as a ubiquitin E3 ligase, playing a pivotal role in mediating the TNFα–NFκB signaling pathway [75]. CUL4A, as an E3 ubiquitin ligase, forms a complex with WDR6 and DDB1 to target UVRAG for ubiquitination and degradation. The degradation of UVRAG blocks the autophagic degradation of p65, thereby maintaining p65 stability and enhancing NF-κB signaling. This process upregulates TNFα expression, promotes MDSC recruitment, and inhibits CD8^+^ T cell infiltration, thereby establishing an immunosuppressive microenvironment in HCC [76]. USP14 stabilizes the IκBα protein in HNSCC cells through its deubiquitinase activity, thereby inhibiting K48-linked ubiquitination and degradation of IκBα. This process prevents TNFα-induced IκBα degradation, suppresses the activation of the NF-κB pathway and reduces TNFα-induced RELA nuclear translocation [73] (Fig. 4b).

#### Epidermal growth factor receptor (EGFR)

EGFR is a critical regulator in multiple cancer types [77, 78], and its signaling is tightly controlled by ubiquitination and deubiquitination processes. Dysregulation of these processes can significantly impact EGFR stability, degradation, and downstream signaling, thus influencing cancer progression and therapeutic responses. FBXW7, a tumor-suppressor E3 ubiquitin ligase, serves as a primary regulator of the ubiquitination and proteasomal degradation of EGFR. In colorectal cancer (CRC), FBXW7 recognizes specific phosphodegron motifs in EGFR, facilitating its ubiquitination and subsequent degradation. Mutations in FBXW7 disrupt this process, stabilizing EGFR and reducing EGF dependency in cancer cells. Consequently, FBXW7 mutations attenuate the efficacy of anti-EGFR therapies, thereby promoting tumor progression [79]. Similarly, CBL, another E3 ubiquitin ligase, regulates EGFR degradation through lysosomal trafficking. In lung adenocarcinoma, cervical cancer, and NSCLC models, CBL targets activated EGFR for ubiquitination and lysosomal degradation, suppressing its signaling. Flotillin-2 has been identified as a regulatory partner of CBL, as its knockdown significantly enhances EGFR ubiquitination and accelerates its degradation, thus inhibiting EGFR-dependent proliferation [80]. In contrast, USP21, a deubiquitinating enzyme, stabilizes EGFR by removing ubiquitin chains and preventing its degradation. In CRC, elevated USP21 expression is associated with increased EGFR stability and tumor progression. Inhibition of USP21 with BAY-805 effectively reduces EGFR levels and suppresses EGFR-mediated cell proliferation, positioning USP21 as a therapeutic target in metastatic CRC [81]. While FBXW7 and CBL primarily promote EGFR degradation, HOIP, as a component of the linear ubiquitin chain assembly complex (LUBAC), serves a distinct function by enhancing EGFR signaling through linear ubiquitination. In epithelial carcinoma and breast cancer models, HOIP is recruited to EGFR via plakophilin 2 (PKP2), leading to linear ubiquitination of NEMO and subsequent activation of the NF-κB pathway [82]. The HOIP inhibitor HOIPIN-8 effectively suppresses EGFR-induced NF-κB activation, highlighting HOIP as a potential target for cancer therapy. These studies provide valuable therapeutic insights for targeting EGFR-driven malignancies.

#### ILs

ILs are key immunomodulatory cytokines within the TME, and their expression, stability, and downstream signaling are tightly regulated by ubiquitination and deubiquitination processes. These PTMs not only govern protein degradation but also influence the intensity and duration of cytokine signaling, shaping both pro- and anti-tumor immune responses. In osteosarcoma, FBXO2, an F-box protein that functions as part of the SKP–Cullin–F-box (SCF) E3 ligase complex, promotes tumor growth by stabilizing the IL-6 receptor (IL-6R), thereby enhancing IL-6/STAT3 signaling. This stabilization requires the FBA domain of FBXO2 for glycoprotein recognition [83]. CRISPR-mediated knockout of FBXO2 significantly impairs STAT3 phosphorylation and downstream gene expression, suppressing tumorigenicity both in vitro and in vivo. Conversely, in cutaneous squamous cell carcinoma (cSCC), the E3 ligase NEDD4L promotes the ubiquitination and lysosomal degradation of GP130, the IL-6 co-receptor, thus downregulating IL-6/STAT3 signaling [84]. Pharmacological inhibition of GP130 (e.g., SC144) synergizes with NEDD4L function, further dampening tumor-promoting inflammation. Upon AKT-mediated phosphorylation, TRAF4 translocates to the nucleus via interaction with 14-3-3θ. In the nucleus, its TRAF domain binds to the transcription factor c-Jun, enhancing IL-8 promoter activity [85]. IL-8 secretion promotes stemness and metastatic dormancy. Blocking nuclear TRAF4 or IL-8 signaling suppresses tumor invasion and dormancy features. These modifications critically shape tumor immunity and represent promising targets for future immunotherapeutic strategies.

Table 1 summarizes the mechanism by which ubiquitination regulates the TIME.

**Table 1:** Mechanism of ubiquitination process regulating TIME

Regulators	Effect on immune function	Inhibitor	Inducer	Immunoregulatory mechanisms	Cancer types	Reference
ARIH1	Facilitates T cell activation	-	ACY738	ARIH1 facilitates the ubiquitination and subsequent degradation of DNA-PKcs, thereby triggering STING pathway activation and facilitating T cell activation.	Melanoma, breast cancer	[47]
Trim21	Promotes CD4^+^ T cell activation	-	-	Trim21 mediates the ubiquitination of CCT2 to promote CD4^+^ T cell activation.	Breast cancer	[48]
Peli1	Reduces CD4⁺ and CD8⁺ T cell infiltration	-	-	Genetic ablation of Peli1 substantially enhances tumor rejection, concomitant with increased infiltration of CD4⁺ and CD8⁺ T cells into the TME.	Melanoma, thymoma	[49]
UBR5	Suppresses CD8⁺ T cell function	-	-	UBR5 facilitates tumor progression primarily via paracrine crosstalk with the immune system, notably by inhibiting CD8⁺ T cell –mediated cytotoxicity.	Breast cancer	[50]
Grail	Suppresses CD8⁺ T cell function	-	TGF-β	In CD8⁺ T cells, Grail modulates TCR and IL‑21R signaling pathways, thereby limiting effector cytokine production and cytolytic activity.	Lymphoma	[51]
Otub1	Controls CD8^+^ T cell and NK cell activation	-	-	Otub1 orchestrates the activation of CD8⁺ T cells and NK cells by acting as a checkpoint in IL‑15–mediated priming.	Melanoma	[52]
BFAR	Suppresses aged CD8⁺ T cell function	iBFAR2	-	BFAR promotes the deubiquitination of JAK2 by activating the deubiquitinase USP39, which in turn suppresses STAT1 phosphorylation.	Melanoma, bladder cancer	[53]
USP7	Reduces CD8⁺ T cell activity	P5091	-	USP7 upregulated FGL1 by deubiquitination of transcription factor PRDM1 and attenuated the CD8^+^ T cell activity.	Liver cancer	[54]
circUSP7	Suppresses CD8⁺ T cell function	-	-	Exosomal circUSP7 enhances immunosuppression in NSCLC by inducing CD8⁺ T cell dysfunction.	NSCLC	[55]
Usp22	Maintains Treg suppressive function	-	-	Usp22, a deubiquitination‑module component of the SAGA chromatin‑modifying complex, functions as a positive regulator by stabilizing Foxp3 expression.	Lymphoma, colon cancer, lung carcinoma and melanoma	[56]
Rnf20	Impairs Treg stability	-	-	The E3 ubiquitin ligase Rnf20 functions as a negative regulator of Foxp3.	Lymphoma, colon cancer, lung carcinoma and melanoma	[56]
USP44	Supports Treg activity		-	USP44 supports Treg activity by preserving FOXP3 protein stability and preventing its degradation.	Melanoma, colon cancer, and thymoma	[57]
TRAF6	Enhances Treg function	-	-	TRAF6 orchestrates FOXP3 localization and enhances Treg function via K63‑linked ubiquitination.	Melanoma, colon cancer	[58]
Cbl-b	Promotes Treg-mediated suppression	-	-	Excessive IL‑2 secretion by Cbl‑b–deficient CD4⁺ T cells or elevated IFNγ production by Cbl‑b–deficient CD8⁺ T cells imparts resistance to Treg‑mediated suppression.	Lymphoma, melanoma	[59, 60]
Cop1	Regulates macrophage infiltration	-	-	Cop1, via the adaptor protein Trib2, governs the protein abundance of TF C/ebpδ. Subsequently, C/ebpδ transcriptionally represses the release of macrophage chemoattractants from cancer cells.	Breast cancer	[63]
FATS	Suppresses antitumor macrophage polarization	-	-	FATS deficiency disrupts the NF‑κB/IκBα negative‑feedback loops, thereby stimulating and prolonging NF‑κB activation and ultimately promoting M1 polarization.	Melanoma and pancreatic tumor	[64]
USP7	Promotes M2-like TAM polarization	P5091, HBX19818, GNE-6776	-	Selective inhibition of USP7 reprogrammed TAMs to M1 macrophages via activation of the p38 MAPK pathway, thereby enhancing CTL‑mediated antitumor responses and ultimately suppressing tumor growth.	Lung cancer	[65]
USP18	Suppresses antitumor macrophage polarization	-	-	USP18 can inhibit macrophage polarization toward an antitumor phenotype by interacting with NEDD4 to affect CSF1R expression. Its deficiency enhances the IFN-I signaling pathway, promoting CSF1R degradation and altering macrophage function.	Melanoma, lymphoma and lung cancer	[66]
USP2	Inhibits TGF-β signaling	-	SGI-1027	USP2 deubiquitinates SMAD7 and directly cleaves Lys 27‑ and Lys 48‑linked polyubiquitin chains.	Glioblastoma	[68]
NEDD4	Augments TGF‑β signal	-	-	NEDD4 augments TGF‑β signal transduction–mediated tumor progression by directly associating with the TGFBR1 and catalyzing K27‑linked ubiquitination at Lys391.	HCC	[69]
CUL4A	Promotes TGF-β/SMAD3 signaling competence	-	-	CUL4A negatively regulates the stability of HUWE1, thereby affecting the ubiquitination status and intracellular localization of SMAD3.	Colorectal cancer	[70]
FBXO38	Antagonizes TGF‑β	-	-	FBXO38 antagonizes TGF‑β–induced downregulation of IL‑15R expression by attenuating Smad2/3 signaling and sustaining Eomes expression.	Lymphoma, melanoma, colon adenocarcinoma	[71]
TRIM55	Inactivates the TGFβ/Smad signaling pathway	-	-	TRIM55 attenuates the interaction between NF90 and the mRNAs of HIF1α and TGF‑β2, thereby inactivating the HIF1α/VEGF and TGFβ/Smad signaling pathways.	HCC	[72]
CUL4A	Promotes TNFα signaling	-	-	CUL4A ubiquitinates and degrades UVRAG, blocking the autophagic degradation of p65 and enhancing NF-κB signaling pathway activity.	HCC	[76]
USP14	Reduces TNFα-induced RELA nuclear translocation	b-AP15, IU1-47	-	USP14 inhibits the K48-linked ubiquitination and degradation of IκBα, suppresses NF-κB pathway activation, and reduces TNFα-induced RELA nuclear translocation.	Head and neck squamous cell carcinoma	[73]
FBXW7	Promotes EGFR degradation	-	-	FBXW7 recognizes specific phosphodegron motifs in EGFR, promoting its ubiquitination and subsequent degradation.	Colorectal Cancer	[79]
CBL	Promotes EGFR degradation	-	-	CBL promotes EGFR ubiquitination and directs it for lysosomal degradation, thereby suppressing EGFR signaling.	NSCLC, Cervical cancer	[80]
USP21	Prevents EGFR degradation	BAY-805	-	USP21 removes ubiquitin chains from EGFR, preventing its degradation and enhancing EGFR signaling.	Colorectal Cancer	[81]
HOIP	Promotes EGFR-driven NF-κB signaling	HOIPIN-8	-	HOIP is recruited to EGFR via PKP2, leading to linear ubiquitination of NEMO and subsequent NF-κB pathway activation.	Breast cancer	[82]
FBXO2	Stabilizes IL-6R	-	-	FBXO2 promotes tumor growth by stabilizing the IL-6R, thereby enhancing IL-6/STAT3 signaling.	Osteosarcoma	[83]
NEDD4L	Downregulates IL-6/STAT3 signaling	-	-	NEDD4L promotes the ubiquitination and lysosomal degradation of GP130, thus downregulating IL-6/STAT3 signaling.	Skin cancer	[84]
TRAF4	Enhances IL-8 promoter activity	-	-	TRAF4 binds transcription factor c-Jun, enhancing IL-8 promoter activity.	Breast cancer, colon cancer, glioma	[85]

*TIME* tumor immune microenvironment, *ARIH1* ariadne RBR E3 ubiquitin‑protein ligase 1, *TME* tumor microenvironment, *UBR5* ubiquitin protein ligase E3 component n‑recognin 5, *TGF-β* transforming growth factor-β, *IL‑21R* IL‑21 receptor, *USP* ubiquitin-specific protease, *PRDM1* PR domain zinc finger protein 1, *NSCLC* non-small cell lung cancer, *Treg* regulatory T, *RNF* Ring finger protein, *FOXP3* forkhead box P3, *TRAF6* TNF receptor‑associated factor 6, *IL* interleukin, *FATS* fragile site-associated tumor suppressor, *NEDD4* neural precursor cell expressed developmentally downregulated 4, *HCC* hepatocellular carcinoma, *TNFα* tumor necrosis factor α, *EGFR* epidermal growth factor receptor

## Ubiquitination process mediates tumor immune evasion

### Immune checkpoints

#### PD-1

PD‑1 (CD279) expression increases upon T cell activation, facilitating interactions with PD‑L1 (CD274/B7‑H1) and PD‑L2 (CD273/B7‑DC) on tumor and immune cells within the TME. Through this engagement, T cell activity is compromised, enabling tumors to evade immune surveillance [6, 86]. Research on PD-1/PD-L1 modulation in cancer immunotherapy has revealed novel intervention targets and therapeutic strategies aimed at improving clinical outcomes [87, 88]. Recently, multiple studies have reported that PD-1 is regulated at the posttranslational level, particularly via ubiquitin-related pathways.

**Fig. 5:**
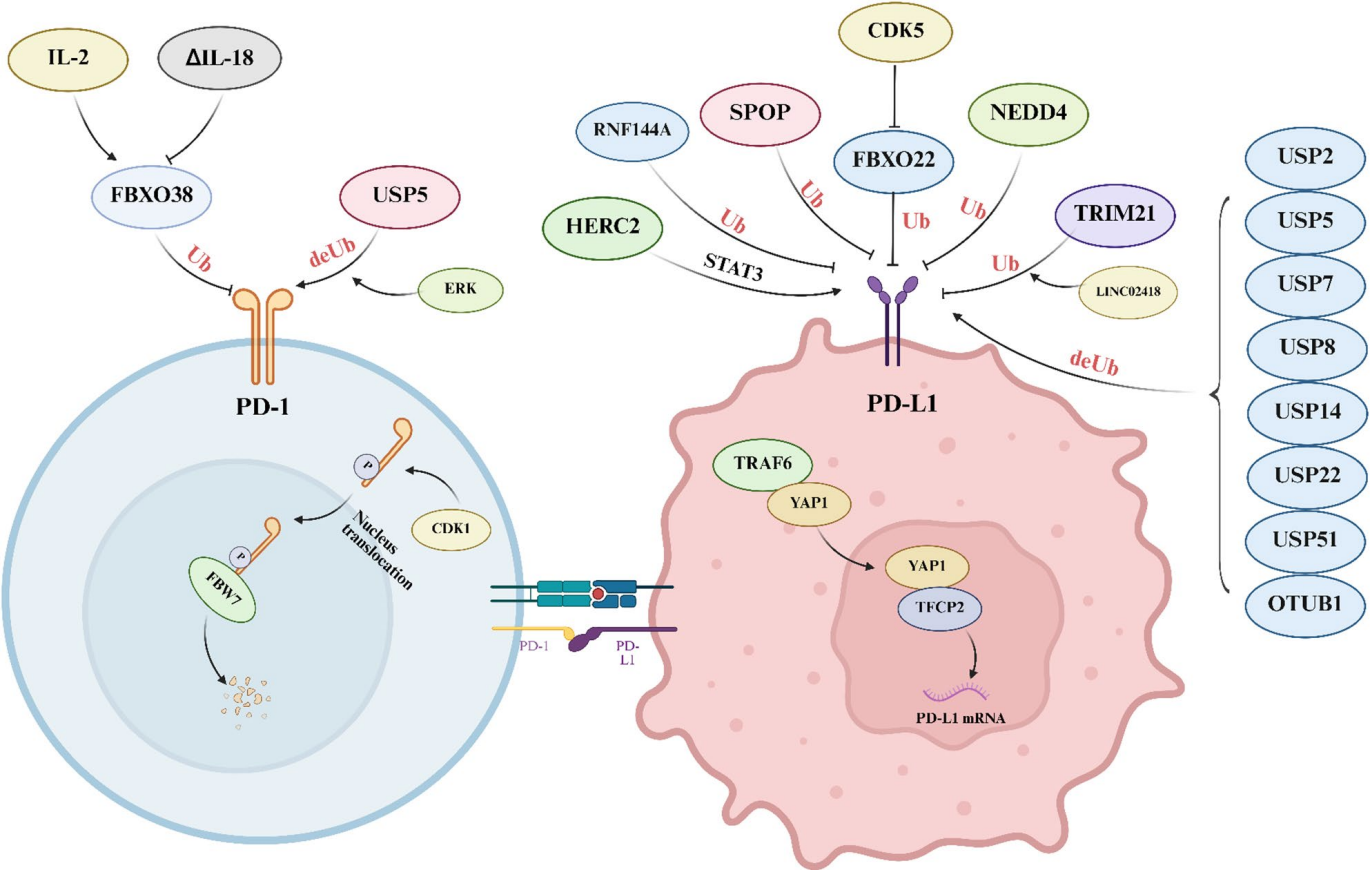
The ubiquitination process regulates the expression of PD-1 and PD-L1. *PD-1* programmed cell death protein 1, *PD-L1* programmed cell death ligand 1, *IL* interleukin, *USP* ubiquitin-specific protease, *Ub* ubiquitin, *CDK* cyclin-dependent kinase, *RNF* Ring finger protein, *SPOP* speckle type POZ protein, *NEDD4* neural precursor cell expressed developmentally downregulated 4, *TRAF6* TNF receptor‑associated factor 6

FBXO38 is an E3 ligase for PD-1 that catalyzes Lys48-linked polyubiquitination and subsequent proteasomal degradation. Conditional deletion of *Fbxo38* in T cells results in elevated PD-1 levels in tumor-infiltrating T cells, leading to accelerated tumor progression. Anti-PD-1 therapy normalizes the effects of FBXO38 deficiency on tumor growth, indicating that PD-1 is the primary target of FBXO38 in T cells [21]. Studies have shown that the administration of IL-2 can rescue *Fbxo38* transcription in tumor-infiltrating T cells, whereas ΔIL-18 downregulates *Fbxo38* transcription levels, reducing PD-1 ubiquitin-mediated degradation and thereby facilitating immune evasion [21, 89]. Another study revealed that FBW7 also serves as an E3 ligase for PD‑1, catalyzing Lys48‑linked polyubiquitination at the Lys233 residue. Cotargeting FBW7 drives PD-1 degradation and augments antitumor immunity in vivo. Additionally, cyclin-dependent kinase 1 (CDK1)‑mediated phosphorylation of Ser261 primes PD‑1 for nuclear translocation and subsequent binding to FBW7 [90]. USP5 deubiquitinates and stabilizes PD‑1. Extracellular signal‑regulated kinase (ERK) phosphorylates PD‑1 at Thr234, increasing its binding to USP5. T cell–specific *Usp5* knockout increases effector cytokine production and slows tumor growth. Combining USP5 inhibition with trametinib or anti‑CTLA‑4 therapy synergistically suppresses tumor progression [91] (Fig. 5).

#### PD-L1

PD-L1 is a prominent immunosuppressive molecule that enables tumor cells to evade immune surveillance, making it a major target in cancer immunotherapy. Elevated expression of PD-L1 on tumor cells disrupts T cell–mediated cytotoxicity via interaction with PD-1. Understanding the regulatory mechanisms controlling PD-L1 expression, including PTMs, is essential for enhancing the efficacy of PD-L1 blockade in cancer therapy.

CRLs constitute a principal family of E3 ubiquitin ligases that catalyze the ubiquitination and degradation of diverse cellular regulators, and the speckle type POZ protein (SPOP) serves as the substrate‑recognition adaptor within the CRL3 complex [92]. Multiple studies have confirmed that SPOP participates in the degradation of PD-L1 mediated by the proteasome pathway [92–95]. FBXO22 belongs to the F-box protein family, which constitutes a component of the SCF ubiquitin E3 ligase complex [96]. FBXO22 facilitates the ubiquitination and degradation of PD-L1, thereby increasing the sensitivity of NSCLC cells to DNA damage. Inhibition of CDK5 or a reduction in its expression increases FBXO22 levels, decreases PD-L1 levels, and further enhances the cells sensitivity to DNA damage [97]. The TRIM family and TRAF family are also E3 ubiquitin ligases containing RING domains. Studies have shown that TRIM21 can mediate the ubiquitination of PD-L1 and that this ubiquitination process can be enhanced by LINC02418 [98]. TRAF6 is an essential regulator of PD-L1, as it stabilizes YAP1 through K63 poly-ubiquitination, which in turn promotes the assembly of the YAP1/TFCP2 transcriptional complex and upregulates PD-L1 gene expression [99]. The HECT family is a group of important E3 ubiquitin ligases, and several of its members have been implicated in the regulation of PD-L1. HERC2 is an E3 ligase that contains a HECT domain. HERC2 promotes stemness in HCC cells and PD-L1–mediated immune evasion, a process linked to activation of the STAT3 pathway during the inflammation‑to‑cancer transition [100]. As a member of the HECT subclass of E3 ubiquitin ligases, NEDD4 is phosphorylated upon FGFR3 activation and drives Lys48‑linked polyubiquitination of PD‑L1 [101]. RBR E3 ubiquitin ligases are recognized as a distinct subclass among the ring finger proteins (RNF) [102]. They feature two conserved zinc‑binding domains flanking a unique zinc‑binding motif and are thought to facilitate ubiquitin transfer via a RING/HECT hybrid‑like mechanism [103]. RNF144A associates with PD‑L1 at the plasma membrane and within intracellular vesicles to drive its poly-ubiquitination and degradation; *Rnf144a* knockout stabilizes PD‑L1 and reduces the population of tumor‑infiltrating CD8⁺ T cells [104] (Fig. 5).

PD-L1 can also be deubiquitinated by various deubiquitinases. Several previous researches have confirmed that USP2, USP5, USP7, USP8, USP14, USP22, and USP51 are the deubiquitinases of PD-L1 [105–114], and that the use of USP inhibitors can inhibit the deubiquitination of PD-L1 mediated by USPs, thus degrading PD-L1. However, in a study on pancreatic cancer, researchers identified USP14 as a post-translational modulator that negatively regulates PD-L1 expression [115]. The seemingly opposing roles of individual DUBs across different tumor types likely reflect context-dependent regulation determined by ubiquitin linkage preference, lysine site usage, subcellular compartmentalization of PD-L1, upstream inflammatory or kinase signaling, and competition among E3 ligases and DUBs within the TIME. In addition, the deubiquitinase OTUB1 can also positively regulate PD-L1 stability by binding its intracellular domain and cleaving K48‑linked ubiquitin chains. Owing to the deubiquitinase activity of OTUB1, this mechanism inhibits ERAD‑dependent degradation of PD‑L1 [116]. Therefore, targeting deubiquitinases may constitute an effective approach to overcome resistance to immunotherapy.

#### Other immune checkpoints

CTLA‑4, a pivotal immune checkpoint molecule abundantly expressed on activated T cells, maintains immune homeostasis while facilitating tumor immune evasion [27, 117]. The CTLA‑4–blocking antibody ipilimumab received approval from the US Food and Drug Administration (FDA) in 2011 for the treatment of malignant tumors, marking the first clinical targeting of an immune checkpoint receptor [118]. Localized in endosomal vesicles, USP8 binds CTLA‑4 and its loss elevates CTLA‑4 ubiquitylation in cancer cells, CD4⁺ T cells, and tumor‑derived exosomes, and knockdown of the USP8 adaptor HD‑PTP reproduces this phenotype [119]. TRAF6, a RING finger–domain E3 ubiquitin ligase, catalyzes Lys63‑linked ubiquitination of CTLA‑4, targeting it for lysosomal degradation; activation of the OX40–TRAF6 axis accelerates CTLA‑4 turnover and represents a promising strategy to enhance T cell–based immunotherapies [118].

Other immune checkpoints regulated by ubiquitination-related enzymes include CD47 and B7-H4. CD47 is an immune checkpoint protein that is overexpressed by tumor cells and can help tumors escape immunity. Studies have shown that the ubiquitin E3 ligase TRIM21 can interact with CD47, mediating the ubiquitination of CD47 K99/102 and the degradation of CD47 [120]. This interaction can be inhibited by c-Src-mediated phosphorylation of CD47 Y288 caused by EGFR activation. B7–H4 is an immune checkpoint molecule that suppresses CD8⁺ T cell activity. USP2a functions as a deubiquitinase by cleaving both K48‑ and K63‑linked ubiquitin chains from B7–H4, thereby diminishing its degradation [121]. Oncogenic EGFR mutants further stabilize B7–H4 through upregulation of USP2a expression.

### MHC

The major histocompatibility complex (MHC) class I and II molecules are critical mediators of the activation and regulation of adaptive immunity, presenting antigens to CD8⁺ and CD4⁺ T cells, respectively [122, 123]. Attenuated antigen presentation by the MHC has been recognized as a potential resistance factor in immune responses. Rigorous regulation of MHC expression is essential for appropriate immune function and efficient T cell activation.

The surface protein sushi domain containing 6 (SUSD6) associates with transmembrane protein 127 (TMEM127) and MHC‑I to form a trimolecular complex that recruits the E3 ubiquitin ligase WWP2, thereby driving MHC‑I ubiquitination and lysosomal degradation and functioning as a principal negative regulator of MHC‑I [124]. The epigenetic regulator ubiquitin‑like with PHD and ring finger domains 1 (UHRF1) is aberrantly expressed and mislocalized to the cytosol in malignant tissues, where it similarly promotes MHC‑I ubiquitination and turnover; the resulting downregulation of MHC‑I suppresses antigen presentation and establishes an immune hostile TME [125]. Moreover, the E3 ubiquitin ligase complex component FBXO11 targets CIITA, the master transcription factor controlling MHC II expression, for degradation—blocking this repressive axis selectively induces MHC II upregulation across diverse acute myeloid leukemia (AML) cell lines [122, 126].

Table 2 summarizes the mechanism of tumor immune escape mediated by ubiquitination process.

**Table 2:** Mechanism of ubiquitination process mediating tumor immune escape

Target	Regulators	Regulation of target	Ubiquitin linkage	Inhibitor	Inducer	Immunoregulatory mechanisms	Cancer types	Reference
PD-1	FBXO38	Down	K48	-	IL-2	FBXO38 catalyzes Lys48‑linked polyubiquitination of PD‑1 at the Lys233 residue, resulting in its degradation.	Melanoma	[21]
	FBXO38	Down	-	IL-18	-	ΔIL‑18 drives immune escape by downregulating *Fbxo38* transcription in CD8⁺ T cells, thereby impairing the ubiquitin‑mediated degradation of PD‑1.	Gallbladder Cancer	[89]
	FBW7	Down	K48	-	Oridonin	FBW7 facilitates K48-linked polyubiquitin chain formation on PD‑1 at lysine residue 233.	NSCLC	[90]
	USP5	Up	K48	EOAI3402143	-	USP5 binds to PD‑1 and facilitates its deubiquitination, thereby enhancing PD‑1 protein stability.	Colorectal cancer	[91]
PD-L1	SPOP	Down	K48	-	-	SPOP mediates the proteasome-dependent degradation of PD-L1.	Breast cancer, colorectal cancer, endometrial cancer	[92–95]
	FBXO22	Down	-	CDK5	-	FBXO22 induces PD‑L1 ubiquitination and subsequent degradation, and CDK5 inhibition or downregulation enhances FBXO22 expression.	NSCLC	[97]
	Trim21	Down	-	-	LINC02418	LINC02418 diminishes PD‑L1 expression by promoting E3 ligase Trim21‑mediated ubiquitination of PD‑L1.	NSCLC	[98]
	TRAF6	Up	K63	Bortezomib	-	TRAF6 preferentially mediates K63‑linked ubiquitin chain formation, thereby regulating PD‑L1 expression.	Melanoma	[99]
	HERC2	Up	-	-	-	HERC2 can enhance PD-L1-mediated immune evasion by activating the STAT3 pathway.	HCC	[100]
	NEDD4	Down	K48	-	FGFR3 activation	NEDD4 is phosphorylated by FGFR3 activation and catalyzes Lys48-linked polyubiquitination of PD-L1.	Bladder cancer	[101]
	RNF144A	Down	-	-	-	RNF144A associates with PD‑L1 at the plasma membrane and within intracellular vesicles, catalyzing its polyubiquitination and subsequent degradation.	Bladder cancer	[104]
	USP7	Up	-	Almac4, P5091	-	USP7 directly binds and stabilizes PD‑L1, whereas USP7 inhibition disrupts PD‑L1/PD‑1 engagement and enhances T cell–mediated cytotoxicity against cancer cells both in vitro and in vivo.	Gastric cancer	[107]
	USP7	Up	-	A11	-	A11 competes with USP7 for PD‑L1 binding, thereby blocking USP7‑mediated deubiquitination and promoting PD‑L1 degradation.	Breast cancer, lung cancer and melanoma	[108]
	USP2	Up	K48	-	-	Thr288, Arg292, and Asp293 within USP2 regulate its association with PD‑L1 by deconjugating K48‑linked polyubiquitin chains at PD‑L1’s Lys270.	Colorectal and prostate cancer	[105]
	USP5	Up	-	-	-	USP5 directly interacts with PD‑L1 and deubiquitinates it, thereby enhancing PD‑L1 protein stability.	NSCLC	[106]
	USP14	Up	-	-	TNF-α	TNF‑α secreted by M1 macrophages augments PD‑L1 abundance through CDK4‑ and USP14‑mediated deubiquitination of PD‑L1.	Head and neck squamous cell carcinoma	[111]
	USP14	Down	K63	-	-	USP14 attenuates PD‑L1 expression by deconjugating K63‑linked ubiquitin chains at the Lys280 residue.	Pancreatic cancer	[115]
	USP8	Up	-	-	-	USP8 inhibits the ubiquitination-dependent proteasomal degradation of PD‑L1.	Pancreatic cancer, NSCLC	[109, 110]
	USP22	Up	K6, K11, K27, K29, K33 and K63	-	-	USP22 directly deubiquitinates PD‑L1, thereby preventing its proteasomal degradation.	Liver cancer, NSCLC	[112, 113]
	USP51	Up	K63	Dihydromyricetin	-	USP51 mediates the deubiquitination of PD‑L1 at lysine residues K280/K281.	NSCLC	[114]
	OTUB1	Up	K48	-	-	OTUB1 associates with the intracellular domain of PD‑L1 and deconjugates K48‑linked ubiquitin chains, thereby obstructing ERAD‑mediated degradation of PD‑L1.	Breast cancer	[116]
CTLA-4	USP8	Up	-	-	-	USP8 deficiency amplifies CTLA‑4 ubiquitylation in cancer cells, CD4⁺ T cells, and cancer cell–derived exosomes.	Melanoma, lung cancer	[119]
	TRAF6	Down	K63	-	OX86	TRAF6 catalyzes Lys63‑linked ubiquitination of CTLA‑4, thereby facilitating its lysosomal degradation.	Melanoma	[118]
CD47	TRIM21	Down	-	c-Src	-	TRIM21 facilitates polyubiquitination of CD47 at Lys99 and Lys102, thereby promoting its degradation.	Glioblastoma	[120]
B7–H4	USP2a	Up	K48/K63	-	-	USP2a serves as a deubiquitinase for B7–H4, removing its K48‑ and K63‑linked ubiquitin chains and thereby attenuating B7–H4 degradation.	Lung adenocarcinoma	[121]
MHC-Ⅰ	WWP2	Down	-	-	-	SUSD6 engages with TMEM127 and MHC‑I to assemble a trimolecular complex that recruits WWP2, thereby facilitating MHC‑I ubiquitination and lysosomal degradation.	AML	[124]
	UHRF1	Down	-	-	-	UHRF1 is aberrantly expressed and improperly localized in the cytoplasm of malignant cells, where it facilitates MHC‑I ubiquitination followed by degradation.	NSCLC	[125]
CIITA	FBXO11	Down	-	-	-	FBXO11 mediates degradation of CIITA, the master transcription factor governing MHC class II expression.	AML	[122, 126]

*PD-1* programmed cell death protein 1, *PD-L1* programmed cell death ligand 1, *NSCLC* non-small cell lung cancer, *USP* ubiquitin-specific protease, *SPOP* speckle type POZ protein, *CDK* cyclin-dependent kinase, *TRAF6* TNF receptor‑associated factor 6, *HCC* hepatocellular carcinoma, *NEDD4* neural precursor cell expressed developmentally downregulated 4, *RNF* Ring finger protein, *TNFα* tumor necrosis factor α, *CTLA-4* cytotoxic T-lymphocyte-associated protein 4, *MHC* major histocompatibility complex, *SUSD6* sushi domain containing 6, *TMEM127* transmembrane protein 127, *UHRF1* ubiquitin‑like with PHD and ring finger domains 1, *AML* acute myeloid leukemia

## Crosstalk between ubiquitination and other PTMs in tumor immunity

**Fig. 6:**
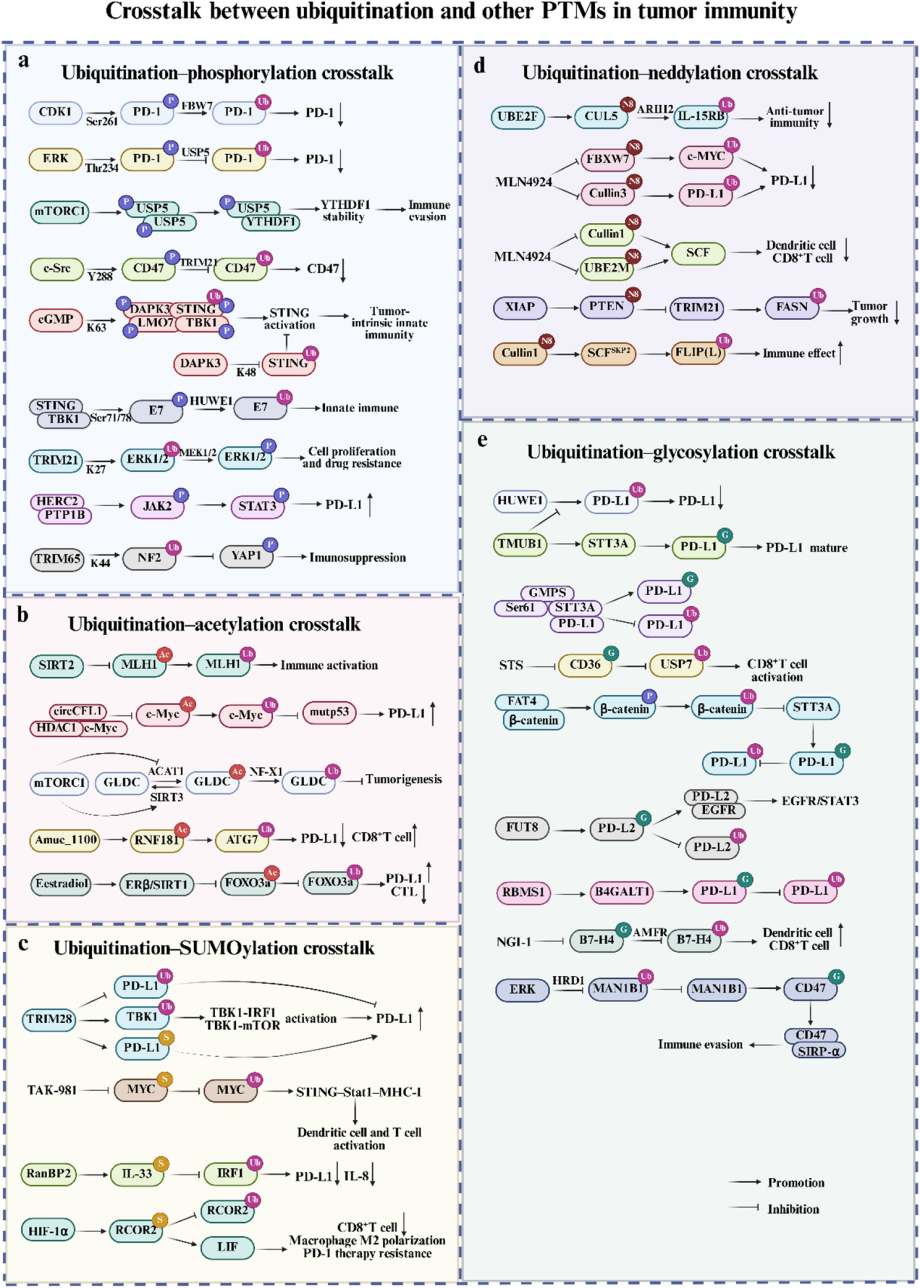
Ubiquitination crosstalk with (**a**) phosphorylation, **b** acetylation, **c** SUMOylation, **d** neddylation, and **e** glycosylation to regulate tumor immunity. *CDK* cyclin-dependent kinase, *PD-1* programmed cell death protein 1, *PD-L1* programmed cell death ligand 1, *ERK* extracellular signal‑regulated kinase, *USP* ubiquitin-specific protease, *GLDC* glycine decarboxylase, *RNF* Ring finger protein, *IL* interleukin, *SCF* SKP–Cullin–F-box, *STS* short-term starvation, *TMUB1* transmembrane and ubiquitin-like domaincontaining protein 1, *EGFR* epidermal growth factor receptor, *HRD1* HMG-CoA reductase degradation 1

Tumor immunity is orchestrated by a complex and dynamic network of PTMs that fine-tune protein function in time and space. Different PTMs can influence one another by altering protein conformation, subcellular localization, and enzyme accessibility. Among these, ubiquitination plays an important role, serving both as a regulatory hub and as a downstream effector of other PTMs such as phosphorylation, acetylation, SUMOylation, neddylation, and glycosylation. Such crosstalk is dynamic and context-dependent, constituting an additional regulatory layer that enables tumor and immune cells to adapt to environmental and therapeutic pressures (Fig. 6). Understanding how ubiquitination communicates with other PTMs has therefore emerged as a new frontier for deciphering immune regulation and resistance mechanisms in cancer immunotherapy.

### Ubiquitination–phosphorylation crosstalk

The interplay between ubiquitination and phosphorylation is one of the most extensively characterized forms of PTM crosstalk (Fig. 6a). Phosphorylation often serves as a molecular switch that determines whether a substrate can be recognized by specific E3 ubiquitin ligases. As mentioned above, the degradation of PD-1 by E3 ligases like FBW7 is contingent upon its phosphorylation. CDK1-mediated phosphorylation of PD-1 at Ser261 is essential for its recognition by FBW7, leading to K48-linked polyubiquitination and degradation [90]. Similarly, ERK-mediated phosphorylation of PD-1 at Thr234 enhances its binding to the deubiquitinase USP5, stabilizing PD-1 [91]. mTORC1-mediated phosphorylation can also promote the dimerization of USP5, enabling it to bind to and stabilize YTHDF1, thereby facilitating cancer immune evasion [127]. In EGFR-activated tumor cells, c-Src–mediated phosphorylation of CD47 at Y288 disrupts its interaction with the E3 ligase TRIM21, thereby preventing K99/102 ubiquitination and degradation [120]. Death-associated protein kinase 3 (DAPK3) exemplifies phosphorylation–ubiquitination crosstalk by suppressing K48-linked ubiquitination and degradation of STING while promoting its K63-linked ubiquitination and TBK1 interaction upon cGAMP stimulation, thereby activating the STING pathway and enhancing anti-tumor immunity [128]. In cervical cancer, TBK1 phosphorylates HPV16/18 E7 oncoproteins at Ser71/Ser78, facilitating HUWE1-mediated ubiquitination and proteasomal degradation of E7, thereby relieving E7-mediated inhibition of the STING pathway and restoring antitumor immunity [129].

Conversely, ubiquitination can regulate kinase stability and activity, thereby modulating major immune signaling cascades. In pituitary adenomas, TRIM21 promotes cell proliferation and drug resistance by mediating K27-linked ubiquitination of ERK1/2, which enhances its interaction with MEK1/2 and facilitates ERK1/2 phosphorylation [130]. HERC2 interacts with the ER-resident phosphatase PTP1B and restricts its translocation to the plasma membrane, thereby relieving its inhibitory effect on JAK2 phosphorylation, activating the JAK2/STAT3 pathway, and upregulating PD-L1 [100]. In HCC, the E3 ligase TRIM65 mediates K44-linked ubiquitination and degradation of NF2, suppressing YAP1 phosphorylation and activating downstream metabolic and immune programs [131].

Within the TIME, this bidirectional regulation shapes the magnitude and duration of immune responses. Hence, the phosphorylation–ubiquitination axis functions as a critical molecular rheostat that tunes immune signaling sensitivity and checkpoint expression. Combining kinase inhibitors with agents targeting the ubiquitin system may yield synergistic effects in reprogramming the TIME.

### Ubiquitination–acetylation crosstalk

Beyond phosphorylation, the interplay between ubiquitination and acetylation constitutes another pivotal regulatory layer in the TIME (Fig. 6b). Both modifications target lysine residues, setting the stage for a dynamic and often competitive relationship that finely controls protein stability and transcriptional activity. For instance, in colorectal cancer, the deacetylase SIRT2 removes acetylation from MLH1 at Lys402/443/461, preventing its ubiquitination and degradation to maintain DNA repair and suppress immune activation; conversely, SIRT2 inhibition promotes MLH1 ubiquitination and degradation, enhancing DNA damage–induced neoantigen production and antitumor immunity [132]. Similarly, in TNBC, circCFL1 functions as a scaffold to enhance the interaction between histone deacetylase 1 (HDAC1) and c-Myc, promoting c-Myc deacetylation and inhibiting its K48-linked ubiquitination and degradation, thereby stabilizing c-Myc, upregulating mutp53 expression, activating the p-AKT/WIP/YAP/TAZ pathway, and inducing PD-L1 expression [133]. In glioma, mTORC1 signaling suppresses glycine decarboxylase (GLDC) K514 acetylation via SIRT3 induction, whereas mTORC1 inhibition allows acetyl-CoA acetyltransferase 1 (ACAT1)-mediated acetylation at K514, which both impairs GLDC enzymatic activity and primes it for NF-X1–mediated K33-linked ubiquitination at K544 and subsequent degradation [134]. In lung adenocarcinoma, the gut microbe–derived protein Amuc_1100 enhances RNF181 expression via promoter acetylation, which in turn promotes ubiquitination and degradation of ATG7, leading to reduced PD-L1 expression and strengthened CD8⁺ T cell–mediated antitumor immunity [135]. Finally, in NSCLC, estradiol activates the ERβ/SIRT1 pathway to deacetylate FOXO3a, promoting its ubiquitination and degradation, thereby suppressing FOXO3a, elevating PD-L1 expression, and impairing CTL cytotoxicity [136]. Collectively, these examples illustrate how ubiquitination and acetylation interact competitively or cooperatively to modulate key oncogenic and immunoregulatory pathways across diverse cancers.

### Ubiquitination–SUMOylation crosstalk

The interplay between ubiquitination and SUMOylation represents a critical regulatory axis that fine-tunes the intensity of immune signaling in the TIME (Fig. 6c). In gastric cancer, the E3 ligase TRIM28 exemplifies this dual-modification control by stabilizing PD-L1 through inhibition of its ubiquitination and enhancement of its SUMOylation, while concurrently promoting K63-linked ubiquitination of TBK1 to activate the TBK1–IRF1 and TBK1–mTOR pathways, further driving PD-L1 transcription and immunosuppression [137]. In KRAS-mutant cancers, inhibition of the SUMOylation cascade via the SUMO-activating enzyme inhibitor TAK-981 promotes MYC degradation through the ubiquitin–proteasome system. This shift toward ubiquitin-mediated degradation activates the STING–Stat1–MHC-I axis and stimulates both dendritic cell and T cell responses, reinforcing immune activation through the suppression of SUMO signaling [138]. In HCC, SUMOylation–ubiquitination crosstalk shapes multiple layers of immune regulation. IL-33, normally enhancing antitumor immunity by promoting ubiquitin-dependent degradation of IRF1 and repressing PD-L1, becomes immunosuppressive upon SUMOylation at Lys54 by RanBP2, which stabilizes IRF1, upregulates PD-L1 and IL-8, and fosters CD8⁺ T cell suppression and M2 macrophage polarization [139]. Similarly, hypoxia-induced HIF-1α upregulates RCOR2 and promotes its SUMOylation, which blocks its ubiquitin-mediated degradation and drives its nuclear translocation. Nuclear SUMOylated RCOR2 enhances LIF transcription, contributing to macrophage M2 polarization, CD8⁺ T cell exhaustion, and PD-1 therapy resistance [140]. Collectively, these studies illustrate that ubiquitination–SUMOylation crosstalk operates as a molecular rheostat controlling the amplitude of immune signaling.

### Ubiquitination–neddylation crosstalk

Neddylation, a ubiquitin-like modification catalyzed by the NEDD8 pathway, mainly activates CRLs—the largest family of E3 ubiquitin ligases—and thereby globally influences ubiquitin-dependent proteostasis and immune signal transduction (Fig. 6d) [141]. In natural killer cells, UBE2F mediates CUL5 neddylation to activate the CRL5 complex, while ARIH2 promotes IL-15RB ubiquitination and degradation, thereby restraining IL-15R signaling. Loss or inhibition of UBE2F or ARIH2 prevents IL-15RB degradation, enhances cytokine production, and boosts NK- and CAR-mediated cytotoxicity [142]. In tumor contexts, neddylation–ubiquitination crosstalk can either suppress or enhance immune responses depending on the substrate and signaling axis. In glioblastoma, neddylation–ubiquitination interplay governs PD-L1 expression and immune evasion. The NEDD8-activating enzyme inhibitor Pevonedistat (MLN4924) suppresses Cullin1–FBXW7 E3 ligase activity, leading to c-MYC accumulation and transcriptional upregulation of PD-L1, while also inhibiting Cullin3-mediated PD-L1 ubiquitination and degradation. This dual mechanism stabilizes PD-L1 and impairs T cell cytotoxicity [143]. Conversely, in malignant pleural mesothelioma, Pevonedistat blocks Cullin-1 and UBE2M neddylation, suppressing SCF complex activity and triggering endoplasmic reticulum stress–dependent immunogenic cell death, which activates CD8⁺T cell–mediated antitumor immunity and enhances cisplatin efficacy [144]. Neddylation also reshapes tumor metabolic–immune coupling. In breast cancer, XIAP-mediated PTEN neddylation promotes its nuclear translocation, where neddylated PTEN suppresses TRIM21-mediated ubiquitination of FASN, enhancing fatty acid synthesis and tumor growth [145]. Moreover, in TRAIL-R2–mediated apoptosis, neddylation-activated Cullin-1, via the SCF^Skp2^ complex, promotes FLIP(L) ubiquitination and degradation, relieving inhibition of TRAIL-R2–induced cell death and enhancing immune effector–mediated cytotoxicity [146]. Collectively, these findings highlight neddylation–ubiquitination crosstalk as a bidirectional regulator of immune signaling intensity. Therapeutically, modulating this interplay offers a promising avenue to balance immune activation and immune tolerance in the TIME.

### Ubiquitination–glycosylation crosstalk

The ubiquitination–glycosylation interplay constitutes a key post-translational regulatory mechanism that governs immune checkpoint stability and tumor immune evasion (Fig. 6e). In multiple cancers, glycosylation frequently protects checkpoint molecules from ubiquitin-dependent degradation, thereby sustaining their immunosuppressive functions. In multiple tumors, transmembrane and ubiquitin-like domaincontaining protein 1 (TMUB1) competes with the E3 ligase HUWE1 to block PD-L1 K281 ubiquitination while recruiting STT3A to enhance its N-glycosylation and maturation, thereby stabilizing PD-L1 and promoting immune escape [147]. Similar mechanisms are observed in HCC, where GMPS bridges the Sect. 61 complex and STT3A to promote PD-L1 glycosylation and suppress ubiquitin-dependent degradation [148], whereas short-term starvation (STS) reverses this process by inhibiting CD36 glycosylation, enhancing USP7 ubiquitination, and relieving T cell exhaustion [149]. In cervical cancer, FAT4 suppresses the β-catenin/STT3A axis, inhibiting PD-L1 glycosylation and promoting its endoplasmic reticulum retention and ubiquitin-mediated degradation, thereby restoring immune recognition [150]. In head and neck squamous cell carcinoma, FUT8-mediated PD-L2 glycosylation stabilizes the PD-L2–EGFR complex, activating EGFR/STAT3 signaling and blocking ubiquitin-dependent lysosomal degradation [151]. Similarly, in TNBC, the RNA-binding protein RBMS1 stabilizes B4GALT1 mRNA, enhancing PD-L1 glycosylation and preventing its ubiquitin-mediated degradation [152], while in immune-cold TNBC, B7-H4 glycosylation prevents AMFR-mediated ubiquitination, with glycosylation blockade by NGI-1 restoring immunogenic cell death and synergizing with chemotherapy and PD-L1 blockade [153]. In bladder cancer, ERK hyperactivation stabilizes the glycosyltransferase MAN1B1 by disrupting its interaction with the E3 ligase HMG-CoA reductase degradation 1 (HRD1), thereby blocking ubiquitin-dependent degradation and enhancing CD47 glycosylation to strengthen its binding with SIRP-α, suppressing phagocytosis [154]. Collectively, these findings highlight glycosylation as a protective modification opposing ubiquitin-mediated degradation, and suggest that disrupting this interplay can restore immune surveillance and potentiate immunotherapy.

## Potential and challenges of targeted ubiquitination regulatory network in tumor immunotherapy

Since ubiquitination-related mechanisms serve as pivotal regulators of tumor immunity, strategies that target ubiquitination have gradually shown significant potential in tumor immunotherapy. In recent years, numerous studies have indicated that by targeting E3 ubiquitin ligases or their regulatory factors, it is possible to effectively modulate the TIME and enhance the efficacy of immunotherapy. For instance, the *Kaempferia parviflora* wall‑derived small molecule 5,7,4′‑trimethoxyflavone (TF) binds to and stabilizes the E3 ubiquitin ligase HRD1, thereby enhancing PD‑L1 ubiquitination in colorectal cancer, lowering PD‑L1 levels, and augmenting T cell–mediated cytotoxicity against tumor cells, ultimately exerting antitumor effects. Moreover, TF synergizes with CTLA-4 antibodies to enhance antitumor immunity. These findings suggest that TF represents a promising small‑molecule immune‑checkpoint modulator for cancer therapy [155]. Additionally, *β*-TrCP is an E3 ubiquitin ligase that plays a crucial role in various human malignancies, notably esophageal squamous cell carcinoma (ESCC). Studies have shown that OTUD6B is an effective deubiquitinase for *β*-TrCP, and that it inhibits ESCC progression via the OTUD6B-*β*-TrCP-SNAIL axis. All‑trans retinoic acid (ATRA) upregulates OTUD6B translation, thereby inhibiting ESCC tumor growth and potentiating the efficacy of anti‑PD‑1 immunotherapy, suggesting that combined ATRA and PD‑1 blockade may offer therapeutic benefit in ESCC [156]. Furthermore, the oncogenic mouse double minute 2 homolog (MDM2), an E3 ubiquitin ligase, drives proteasomal degradation of the tumor suppressor p53. AMG‑232, a selective MDM2 inhibitor, augments T cell–mediated cytotoxicity against tumor cells when combined with anti‑PD‑1 antibody therapy without impairing T cell viability. Thus, targeting MDM2 in tumors exhibiting MDM2 overexpression or gene amplification may represent a precise therapeutic strategy and an alternative means to overcome resistance to immune checkpoint blockade [157]. Tian et al.‘s study confirmed that the antidepressant maprotiline (MAP) can reduce the expression of PD-L1 by targeting the E3 ubiquitin ligase SPOP and that the combination of MAP with anti-CTLA-4 in vivo significantly enhances anti-tumor effects, providing a new option for the clinical treatment of colorectal and lung cancers [158]. Collectively, these studies underscore the significant potential of targeting ubiquitination pathways to enhance tumor immunotherapy and improve clinical outcomes.

In addition to targeting E3 ubiquitin ligases, USPs, which play a key role in the deubiquitination process, are also potential therapeutic targets. Yin‑yang‑1 (YY1) constitutes a critical transcription factor orchestrating tumor cell proliferation, migratory capacity, and epithelial–mesenchymal transition (EMT). USP7 preserves YY1 expression via its deubiquitinase activity. The USP7 inhibitor isorhamnetin (ISO) can inhibit EMT by blocking the deubiquitinase activity of USP7 toward YY1. Researchers have developed an ISO-based nanomedicine, HMSN-ISO@ProA-PD-L1 Ab, which can improve the TIME and inhibit tumor progression, demonstrating the potential of a therapeutic strategy combining USP7 inhibitors and anti-PD-L1 monoclonal antibodies for HCC treatment [159]. Similarly, another study developed engineered radiation-treated cell-released microparticles containing USP7 inhibitors, which can kill tumor cells and reprogram M2 macrophages. This approach improved the survival rate in a mouse model of lung cancer brain metastasis, and when combined with immune checkpoint blockade therapy, the therapeutic efficacy was further enhanced [160]. Zhang et al. discovered through virtual screening and biological evaluation that Rottlerin and Morusin are selective and effective USP22 inhibitors derived from natural compounds [161]. These compounds exhibit significant antitumor activity and have potential applications in cancer therapy. In Lu et al.‘s study, the first natural product, gentiopicroside, was identified as a USP22 inhibitor that can enhance antitumor immunity [162]. Liu et al. combined IU1, a USP14 inhibitor, with IFNα and anti-CTLA-4 treatment, and reported that the combination effectively suppressed tumor growth by blocking the TRIM14/USP14 axis without appreciable toxicity. These results suggest a selective‑autophagy–directed strategy to abrogate PD‑L1–mediated immune evasion in cancer [163]. Studies have demonstrated the potential of targeting USPs with specific inhibitors, either as monotherapies or in combination with immune checkpoint therapies, to enhance antitumor immunity and restrain tumor progression in various cancers.

In recent years, proteolysis-targeting chimera (PROTAC) and molecular glue technologies have emerged as powerful strategies to selectively eliminate oncogenic or immunosuppressive proteins by harnessing the UPS, thereby creating new opportunities for cancer therapy. Mechanistically, PROTACs hijack the intracellular UPS to induce target degradation through formation of a ternary complex composed of a target-binding ligand, an E3 ubiquitin ligase ligand, and a linker [164, 165]. Building on this principle, multiple PD-L1–directed PROTAC strategies have been developed to enhance antitumor immunity. For example, cyclic iRGD peptide–engineered PROTAC nanoparticles (iRP NPs) improved tumor-specific accumulation and enabled efficient PD-L1 degradation, leading to 80.88% tumor regression in an MC38 colon cancer model [166]. Similarly, self-assembled peptide-derived PROTAC nanoparticles (PT-NPs) were designed to achieve precise and durable PD-L1 degradation in targeted tumors [167]. Beyond peptide platforms, a carbon-dot–based PROTAC (CDTAC) was reported to bind PD-L1 and recruit cereblon (CRBN), triggering PD-L1 ubiquitination and proteasomal degradation, with > 99% and > 90% PD-L1 depletion in CT26 and B16-F10 tumor cells, respectively [168]. In addition, checkpoint nano-PROTACs have been integrated with photodynamic therapy to simultaneously drive tumor regression and degrade immunosuppressive proteins, thereby enabling activatable photo-immunotherapy [169]. More designs also extend PROTAC scope to multi-target degradation: a dual-targeting fluorous peptide-based PROTAC (DFP PROTAC) leverages supramolecular self-assembly to induce concurrent degradation of extracellular PD-L1 and cytosolic Bcl-xL via the UPS [170]. These studies highlight rapid innovation in PROTAC architectures—from small molecules to self-assembled nanoformulations—and support PROTAC-based protein degradation as a versatile strategy to potentiate immune checkpoint therapy. Notably, molecular glue mechanisms can also reprogram E3 substrate specificity to enhance innate immunity: small-molecule SPOP inhibitors not only prevented SPOP-mediated STING recognition, but also acted as molecular glues that redirected SPOP toward neo-substrates (e.g., CBX4) for degradation, thereby activating STING signaling, amplifying innate immune responses, and promoting infiltration of immune cells associated with improved anti-PD-1 responsiveness [171], which underscores the versatility of UPS-targeted degradation platforms in reshaping the TIME and overcoming immune evasion.

Despite the encouraging preclinical efficacy of E3 ligase/DUB modulation and UPS-directed degraders, several translational challenges warrant a more cautious interpretation. To date, most E3 ligase- or DUB-targeting agents discussed here remain at the preclinical stage, with limited progression into cancer immunotherapy trials, primarily due to challenges in selectivity, safety windows, and pharmacokinetics. First, target specificity remains a major hurdle, as many E3 ligases and DUBs regulate multiple substrates and pathways, raising the risk that pharmacologic perturbation will produce unintended remodeling of signaling networks and the TIME; notably, the DUB modulator VLX1570 was associated with severe toxicities in a phase I trial, highlighting the challenges of therapeutically targeting broadly interconnected DUBs [172]. Second, because ubiquitination and deubiquitination are core components of global proteostasis, even “on-target” interventions may incur mechanism-based toxicities in normal tissues and immune cells; for example, MDM2 inhibitors such as AMG 232 (KRT-232) have shown dose-limiting cytopenias in clinical studies [173], and milademetan required intermittent dosing to mitigate on-target hematologic toxicities while maintaining activity [174]. Third, pharmacological barriers are non-trivial: PROTACs often suffer from physicochemical properties that limit permeability and oral bioavailability [175], and emerging evidence suggests that tissue distribution and tumor retention can be key determinants of in vivo efficacy [176]; while nanoformulations may improve delivery, they introduce additional translational variables such as scalability, regulatory complexity, and long-term safety assessment [177]. Collectively, future progress will depend not only on identifying immunologically relevant E3 ligases/DUBs, but also on improving degrader/inhibitor selectivity, developing biomarkers to stratify responsive tumors, and establishing exposure–response and safety windows in clinically tractable regimens.

## Conclusions and perspectives

Ubiquitination has evolved from a degradation signal into an important regulatory language of tumor immunity. It acts as a molecular switch controlling immune cell activation, checkpoint turnover, and inflammatory signaling within the TME. The discovery that ubiquitination engages in extensive crosstalk with other PTMs reveals an integrated regulatory network that governs both tumor immune suppression and therapeutic response. This interconnected system allows tumors to fine-tune immune signaling for survival advantage while offering new opportunities for therapeutic intervention.

Future research should prioritize several directions. First, dissecting the spatiotemporal dynamics of PTM crosstalk through advanced proteomics and single-cell technologies will clarify how multiple modifications co-regulate immune proteins in real time. Second, the development of highly selective modulators of E3 ligases and deubiquitinases is essential to achieve precise therapeutic targeting with minimal toxicity. Potential strategies to improve immune specificity may include immune cell–directed delivery systems, tumor microenvironment–activated prodrugs, conditional or context-dependent degraders responsive to hypoxia or inflammatory cues. Third, rational combination strategies—such as pairing ubiquitination inhibitors with checkpoint blockade, metabolic regulators, or DNA damage agents—could synergistically enhance immune responsiveness. Finally, integrating PTM-based biomarkers into clinical trials will enable patient stratification and prediction of immunotherapy outcomes.

In summary, unraveling and therapeutically manipulating the ubiquitination-centered PTM network will pave the way for next-generation precision immunotherapies. By bridging protein modification biology with clinical immuno-oncology, future strategies may overcome immune resistance and transform immunologically “cold” tumors into “hot” ones, ultimately improving survival outcomes for cancer patients.

## Data Availability

No datasets were generated or analysed during the current study.

## Abbreviations

TIME: Tumor immune microenvironment
ICIs: Immune checkpoint inhibitors
PD-1/PD-L1: Programmed cell death protein 1/ programmed cell death ligand 1
CTLA-4: Cytotoxic T-lymphocyte-associated protein 4
TME: Tumor microenvironment
PTM: Post-translational modification
UPS: Ubiquitin-proteasome system
E1: Ubiquitin-activating enzymes
E2: Ubiquitin-conjugating enzymes
E3: Ubiquitin ligases
RING: Really Interesting New Gene
HECT: Homologous to E6-AP Carboxyl Terminus
RBR: RING-Between-RING
DUB: Deubiquitinating enzyme
CAR-T: Chimeric antigen receptor T
irAE: Immune-related adverse event
Treg: Regulatory T
MDSC: Myeloid-derived suppressor cell
ARIH1: Ariadne RBR E3 ubiquitin‑protein ligase 1
UBR5: Ubiquitin protein ligase E3 component n‑recognin 5
IL: Interleukin
IL‑21R: IL‑21 receptor
NK: Natural killer
USP: Ubiquitin-specific protease
PRDM1: PR domain zinc finger protein 1
NSCLC: Non-small cell lung cancer
SH2: Src homology region 2
SHP2: SH2-containing protein tyrosine phosphatase 2
FOXP3: Forkhead box P3
TGF-β: Transforming growth factor-β
TRAF6: TNF receptor‑associated factor 6
TNBC: Triple-negative breast cancer
FATS: Fragile site-associated tumor suppressor
NEDD4: Neural precursor cell expressed developmentally downregulated 4
TAM: Tumor-associated macrophage
HCC: Hepatocellular carcinoma
TGFBR1: TGF-β type I receptor
CRL4: Cullin‑RING E3 ligases 4
TINK: Tumor-infiltrating NK
TNFα: Tumor necrosis factor α
TNFR: TNF receptor
EGFR: Epidermal growth factor receptor
CRC: Colorectal cancer
LUBAC: Linear ubiquitin chain assembly complex
PKP2: Plakophilin 2
SCF: SKP–Cullin–F-box
cSCC: Cutaneous squamous cell carcinoma
CDK1: Cyclin-dependent kinase 1
ERK: Extracellular signal‑regulated kinase
SPOP: Speckle type POZ protein
RNF: Ring finger protein
FDA: Food and Drug Administration
MHC: Major histocompatibility complex
SUSD6: Sushi domain containing 6
TMEM127: Transmembrane protein 127
UHRF1: Ubiquitin‑like with PHD and ring finger domains 1
AML: Acute myeloid leukemia
DAPK3: Death-associated protein kinase 3
HDAC1: Histone deacetylase 1
GLDC: Glycine decarboxylase
ACAT1: Acetyl-CoA acetyltransferase 1
TMUB1: Transmembrane and ubiquitin-like domaincontaining protein 1
STS: Short-term starvation
HRD1: HMG‑CoA reductase degradation protein 1
TF: Trimethoxyflavone
ESCC: Esophageal squamous cell carcinoma
ATRA: All‑trans retinoic acid
MDM2: Mouse double minute 2 homolog
MAP: Maprotiline
YY1: Yin‑yang‑1
EMT: Epithelial–mesenchymal transition
ISO: Isorhamnetin
PROTAC: Proteolysis-targeting chimera
iRP NPs: iRGD peptide–engineered PROTAC nanoparticles
PT-NPs: Peptide-derived PROTAC nanoparticles
CDTAC: Carbon-dot–based PROTAC
CRBN: Cereblon
DFP: PROTAC Dual-targeting fluorous peptide–based PROTAC
Ub: Ubiquitin
ATP: Adenosine triphosphate
AMP: Adenosine monophosphate
DD: Death domain
IKK: Inhibitor of kappa B kinase
IκBα: Inhibitor of kappa B alpha
